# Use of three‐dimensional (3D) optical flow method in mapping 3D anatomic structure and tumor contours across four‐dimensional computed tomography data

**DOI:** 10.1120/jacmp.v9i1.2738

**Published:** 2008-02-05

**Authors:** Geoffrey Zhang, Tzung‐Chi Huang, Thomas Guerrero, Kang‐Ping Lin, Craig Stevens, George Starkschall, Ken Forster

**Affiliations:** ^1^ Department of Radiation Oncology, H. Lee Moffitt Cancer Center University of South Florida Tampa Florida; ^2^ Department of Radiation Oncology University of Texas MD Anderson Cancer Center Houston Texas; ^3^ Chung–Yuan Christian University Chung Li Taiwan

**Keywords:** 3D optical flow, deformable image registration, 4D treatment planning, radiotherapy

## Abstract

A three‐dimensional (3D) optical flow program that includes a multi‐resolution feature has been developed and applied to 3D anatomic structure and gross tumor volume (GTV) contour mapping for four‐dimensional computed tomography (4D CT) data. The present study includes contour mapping for actual CT data sets from 3 patients and also for a thoracic phantom in which the displacement for each voxel was known. Of the CT data sets for the actual patients, one set was used to map lung and GTV contours over all respiration phases, and the other two were studied using only the end inspiration and end expiration phases, in which the displacements between phases were the largest. Including the residual motion in the 4D CT data and motion from table shaking, the optical flow calculation agrees with the known displacement to within 1 mm. Excluding errors not introduced by the optical flow algorithm, agreement for a displacement magnitude of 24 mm can be within 0.1 mm. The mapped contours in 4D CT images of lungs, liver, esophagus, GTV, and other structures for actual patients were acceptable to clinicians. The 3D optical flow program is a good tool for contour mapping of anatomic structure and tumor volume across 4D CT scans.

PACS numbers: 87.55.D‐, 87.59.bd

## I. INTRODUCTION

Tumors in the thorax and the abdomen—such as those of lung, esophagus, and liver—move as patients breathe. Because of the respiratory motion, the dose delivered to the tumors in radiotherapy treatments may not be the same as planned.^(1)^ Although many techniques such as gating^(2)^ and active breathing control^(3)^ are currently used clinically to reduce the effect of respiratory motion on dose delivery accuracy, the problems of dose accuracy and patient comfort during treatments are not yet satisfactorily solved. To overcome tumor motion caused by respiration, four‐dimensional (4D) treatment planning and dose delivery has been proposed.^(4)^ Eventually, to consider the doses to the tumor and to the surrounding structures with regard to respiration phases, a treatment plan would include multiphase subplans that are based on computed tomography (CT) scans of a patient's various respiratory levels. Each subplan is either an optimized plan for a specific respiration phase or an adaptive plan with the treatment beams following the moving target. A tracking system detects the patient's respiratory phase during treatment, and the treatment system determines the subplan to use based on the tracking system's feedback. To apply 4D treatment planning and dose delivery, 4D CT scans have been acquired.^(5)^

In planning for 3D conformal radiotherapy or intensity‐modulated radiotherapy, normal anatomic structures and tumor volumes have to be contoured to define treatment fields and to calculate dose distributions. In conventional 3D treatment planning, normal anatomic structures need be contoured only once. In 4D treatment planning, which essentially consists of multiple 3D plans (one per phase), many sets of CT scan data are involved. In the thorax, not only tumors but also normal anatomic structures change with respiratory motion. One set of contours for the anatomic structures and the tumor volumes would not fit all of the images involved. Manually contouring these structures for 4D treatment planning would take a considerable multiple of the time required for traditional 3D treatment planning. To reduce the clinician's contouring time, we propose using a deformable image registration algorithm based on an optical flow method to assist the contouring process.

Optical flow takes brightness constancy as its basic assumption. The optical flow algorithm maps images elastically, voxel‐to‐voxel. The basic principle of the method used in the present study is that a 3D CT image data set acquired at one phase of the respiratory cycle is deformed to the data set acquired at another phase. The motion field is then applied to contours delineated on the original image data set to yield contours on the second data set.

The optical flow algorithm differs from other methods of image‐deformable registration in its ease of use and precision in mapping structures of interest. No user intervention is required to select matching control points, and the entire image volume is mapped in one step.

Although many good two‐dimensional (2D) optical flow programs have been developed and are available, most deformable image registration applications in radiotherapy for cancer patient treatments^(6)^—including the 3D contouring study discussed in the present paper—require the use of a 3D optical flow program. To that end, a 3D optical flow program was implemented and validated based on an extension to Horn and Schunck's gradient‐based algorithm.^(^ ^7^ ^,^ ^8^ ^)^ The gradient‐based algorithm was chosen for considerations of accuracy.

## II. MATERIALS AND METHODS

### A. 4D image data

To test the algorithm, we used various 4D CT image data sets. One set came from a thoracic phantom developed at the M.D. Anderson Radiological Physics Center (RPC).^(9)^ The phantom was placed on a table that allowed for programmable one‐dimensional (1D) motion. To simulate respiratory motion, the 1D motion was set in the superior–inferior direction at 18 cycles per minute, with a motion distance of 17.5 mm.

Three other 4D CT sets were acquired from patients involved in an institutional review board–approved clinical protocol. The CT data from the 3 patients were used in contour mapping for anatomic structures and tumor volumes.

**Figure 1:**
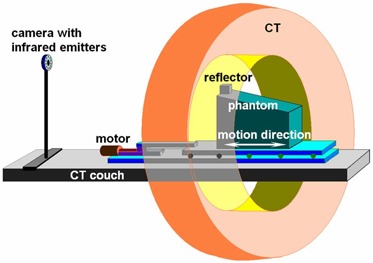
Setup of the four‐dimensional computed tomography (4D CT) scan for the phantom motion study. The Real‐time Position Management system from Varian Medical Systems (Palo Alto, CA) was used in the 4D CT scan. The reflector placed atop the phantom reflects the infrared light from the infrared light emitters (integrated with the video camera), and the video camera records the signal with time stamps. The acquired CT data are then binned into multiple respiratory phases according to the time of the infrared light signal.

All data sets were acquired on a commercial multislice helical CT scanner (MX8000 IDT: Philips Medical Systems, Cleveland, OH). The voxel size in all 4D CT images was 1.0×1.0×3.0 mm. To capture the images, a video camera monitored the marks attached to the patient's chest or the anterior of the phantom to provide gating signals. The 4D data were sorted using amplitude mode. Fig. 1 shows the setup of the phantom 4D imaging. After data acquisition was complete, the system reconstructed the CT data and binned those data into various respiratory phases according to the associated gating signals. Usually, 10 phases were reconstructed per respiratory cycle.

On one of the 4D data sets, a physician contoured anatomic structures and tumor volumes on the end expiration phase. The motion fields calculated by image registration were then used to elastically map the original contours to all the other respiratory phases.

### B. 3D optical flow

Like most motion‐estimation algorithms based on image intensities, the basic assumption of the 3D optical flow algorithm used here is that the intensity of any infinitesimal volume changes little with time, indicating that the material is incompressible. A second constraint, the velocity smoothness constraint, is also included into the algorithm.^(7)^ Applying variational calculus, the algorithm uses three Gauss–Seidel iterations to calculate the three velocity components:

(1) $$\begin{array}{l} v_{x}^{\left(n+1\right)}=v_{x}^{\left(n\right)}-\frac{\partial f}{\partial x}\frac{v_{x}^{\left(n\right)}\frac{\partial f}{\partial x}+v_{y}^{\left(n\right)}\frac{\partial f}{\partial y}+v_{z}^{\left(n\right)}\frac{\partial f}{\partial z}+\frac{\partial f}{\partial t}}{\alpha^{2}+{\left(\frac{\partial f}{\partial x}\right)}^{2}+{\left(\frac{\partial f}{\partial y}\right)}^{2}+{\left(\frac{\partial f}{\partial z}\right)}^{2}}\\ v_{y}^{\left(n+1\right)}=v_{y}^{\left(n\right)}-\frac{\partial f}{\partial y}\frac{v_{x}^{\left(n\right)}\frac{\partial f}{\partial x}+v_{y}^{\left(n\right)}\frac{\partial f}{\partial y}+v_{z}^{\left(n\right)}\frac{\partial f}{\partial z}+\frac{\partial f}{\partial t}}{\alpha^{2}+{\left(\frac{\partial f}{\partial x}\right)}^{2}+{\left(\frac{\partial f}{\partial y}\right)}^{2}+{\left(\frac{\partial f}{\partial z}\right)}^{2}}\\ v_{z}^{\left(n+1\right)}=v_{z}^{\left(n\right)}-\frac{\partial f}{\partial z}\frac{v_{x}^{\left(n\right)}\frac{\partial f}{\partial x}+v_{y}^{\left(n\right)}\frac{\partial f}{\partial y}+v_{z}^{\left(n\right)}\frac{\partial f}{\partial z}+\frac{\partial f}{\partial t}}{\alpha^{2}+{\left(\frac{\partial f}{\partial x}\right)}^{2}+{\left(\frac{\partial f}{\partial y}\right)}^{2}+{\left(\frac{\partial f}{\partial z}\right)}^{2},} \end{array}$$

where *f* denotes the image intensity at a point (*x, y, z*) at time *t*; and $v_{x}=dx/dt,\;v_{y}=dy/dt$, and $v_{z}=dz/dt$ are originally defined as the three components of the velocity that describe the spatial change rate of the voxel with respect to time. These are the three components of the spatial displacement of the voxel between the two image sets involved in an optical flow calculation. The initial $v_{x}^{\left(0\right)},v_{y}^{\left(0\right)},\;and\;v_{z}^{\left(0\right)}$ are set to 0. The intensity gradient components in equation 1 are calculated beforehand by averaging a forward difference in a finite volume. Term α is interpreted as a weighting factor. The value of α is set empirically at 5.

An optical flow calculation requires, as input, a source image and a target image. To assemble the target image, the output from the calculation provides a 3D‐motion field data file equal to each voxel's displacement and an estimated image.

### C. Multi‐resolution feature

Originally, the optical flow method could handle only very small displacements (less than 1 voxel difference), limiting its application. That problem was solved by implementing a multi‐resolution technique. With a larger voxel size at a lower resolution, the magnitude of the displacement between two image sets declines.

To show how the multi‐resolution feature works, here is a 2D example: Two CT image slices each contain 512×512 pixels with resolution of 1×1 mm per pixel. A displacement of 4 mm in the lateral direction for a given region in the two CT slices is therefore represented by 4 pixels at the given resolution level. After subsampling of the CT image to 256×256 pixels per slice, the resolution becomes 2×2 mm per pixel. The 4 mm displacement is now represented by 2 pixels. At resolution of 4×4 mm per pixel (128×128 pixels per slice), the displacement becomes just 1 pixel. If the optical flow program is used to register the two slices, the registration can be very accurate in a short calculation time. After this registration at a coarse resolution level, linear interpolation is used to expand the resulting 128×128 displacement matrix to 256×256. The optical flow program resumes the registration at the 256×256 resolution level, starting with the expanded matrix representing the registered and expanded images. The initial displacement at this resolution level is within 1 pixel instead of 2 pixels if the lower‐level registration is lacking. This procedure continues until the finest resolution level is completed. In this example, the initial displacement at the finest resolution is within 1 pixel after the pre‐registration at coarser levels. The registration with multi‐resolution feature is much more accurate and converges to the solution much faster.

When the image set is downsized from 512×512×70 voxels to 256×256×35 voxels, the intensity of every 2×2×2‐pixel unit in the 512×512×70‐voxel image is averaged to become the intensity of 1 pixel in the 256×256×35‐voxel image. Linear interpolation is used to upsize the images.

The multi‐resolution feature implemented in the program is 3D, and thus it can start with fewer CT slices. The registration starts at a user‐specified resolution level that is a 2^*n*th^ multiple of the original resolution and that increases hierarchically until the finest resolution is achieved.

**Figure 2:**
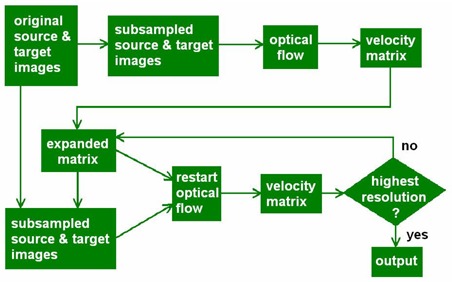
Multi‐resolution flow chart used in the three‐dimensional optical flow program. “Restart optical flow” means to start the registration with the motion field that resulted from the previous resolution level.

Fig. 2 shows a flow chart of the multi‐resolution feature. With the multi‐resolution feature, optical flow is more suitable to radiotherapy image registration applications in which relatively large image changes may often occur. In the present paper, the multi‐resolution feature is used for all contour mapping.

### D. Computation of optical flow

The optical flow calculation usually requires that the computer performing the calculation have a large memory. The images involved in the calculations are in the raw format of 2 bytes per voxel. The memory needed for the source, the target, and the calculated images is outdone by the larger use of memory for the motion fields. For each voxel, a 4‐byte variable in float format is needed to describe each of the three displacement components. In 3D optical flow calculations, the displacement for each voxel needs to be updated after each iteration of velocity calculation, and so a large buffer needs to be assigned for the updating.

**Figure 3:**
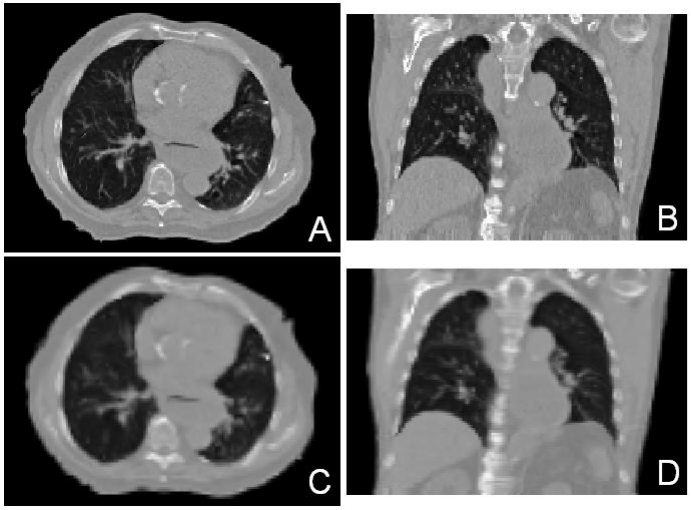
The registered images at the original (2 mm per pixel) and a coarser (4 mm per pixel) resolution are compared. Panels A and B (original resolution) show the transverse and coronal views of the registered images of end expiration to end inspiration. Panels C and D show the same views at the coarser resolution.

To cope with the memory problem, optical flow calculations can be performed at a rougher resolution. Linear interpolation is then used to expand the calculated motion field to the number of voxels found in the original image sets. For example, in changing the resolution to 256×256 pixels from 512×512 pixels per slice, the memory required decreases by a factor of 4. The required calculation time also declines to roughly a quarter of that needed at the higher resolution. The tradeoff is a reduction in precision by a factor of 2 in each of the two directions. Fig. 3 shows a comparison of the registered images of end expiration to end inspiration at various resolutions of a patient's 4D CT data. Panels A and B of Fig. 3 show the registration at the original resolution; panels C and D show the same views at a resolution downgraded by a factor of 2. The blurring effect on panels C and D is caused by the reduced precision of the registration.

The other solution to the memory problem is to calculate in parallel. Parallel calculation is feasible because optical flow calculation involves information change that is locally relative. For example, deformation of liver has no mathematical relationship with deformation of heart. The source and target images can both be divided into two or more sections, with each pair of sections being fed to a separate computer that then performs the relevant optical flow calculation. This sectioning requires a small number of overlapping slices. The results are reassembled with the overlapping parts removed. The calculation time is reduced by a factor roughly equal to the number of sections. Currently, parallel calculation is realized manually. Two sections, each apportioned to a computer with 2 GB of memory, are usually sufficient to solve the memory problem if each image is less than 512×512×150 voxels in size. The difference between the single‐run registration and the parallel registration is minimal, usually less than 0.1 mm on average for a full resolution registration.

During our testing of the program, we varied the convergence criteria to reflect different cost functions. For example, when mutual information was used as the cost function, the calculation stopped if the entropy of the joint histogram varied by less than 1% for 3 iterations. After many tests, our conclusion was that approximately 100 iterations are needed to reach the criterion at the first resolution level, and that fewer iterations are required as the resolution level goes up.

In all the registration calculations in the present study, the number of iterations was set to 100 for registration at the coarsest resolution. This number was gradually reduced to 5 iterations for the run at the finest resolution. In the multi‐resolution registration process, 4 resolution levels were typically used.

## III. RESULTS AND DISCUSSION

We applied optical flow in structure contour mapping of the 4D CT scan data of an RPC thoracic phantom on a motion table that moved in one direction repetitively. In this case, only 1D translation was involved. Contours of lungs, heart, and spinal cord were mapped from the contoured image to all other images.

**Figure 4:**
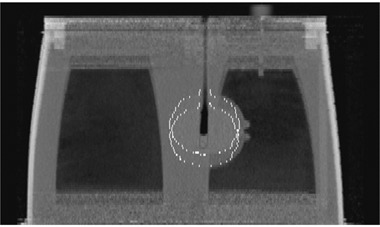
Overlaid coronal view of the original and mapped heart contour images of the thoracic phantom developed at the M.D. Anderson Radiological Physics Center. The original contour is on the end inspiration phase (inferior) and the mapped contour is on the end expiration phase (superior). The displacement between the two extreme phases is 24 mm. The contour follows the phantom motion precisely.

Fig. 4 shows an example of heart contour mapping in the coronal view. The original contour was drawn on the end inspiration phase (inferior contour in the figure), and that contour was then mapped to the end expiration phase (superior contour in the figure). The coronal view of the two phases with the contours was then overlaid, and the displacement of each voxel inside the contours was calculated using the motion field.

**Figure 5:**
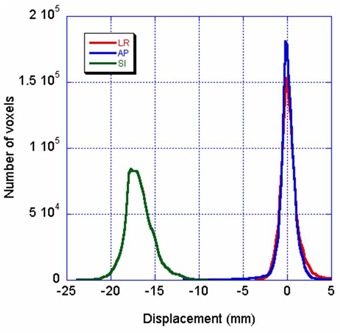
Histogram of the calculated displacement in contoured right lung based on four‐dimensional computed tomography scans of the thoracic phantom developed at the M.D. Anderson Radiological Physics Center. The motion distance was 17.5 mm in the superior–inferior (SI) direction. The calculated SI displacements peaked at 17.5 mm, with a full‐width half‐maximum (FWHM) of about 2.7 mm; the lateral (LR) and anterior–posterior (AP) displacements peaked at 0 mm, with a FWHM of about 1.4 mm.

Fig. 5 shows the histogram of the right lung displacement. The estimated root‐mean‐square (RMS) superior‐inferior displacement of the contoured volume agrees very well with the known displacement. The estimated displacement for the right lung was 16.8±1.5 mm; the displacement on the two CT scans was 17.5 mm.

The RMS superior‐inferior displacement was determined by

(2) $$Displacement{\left(SI\right)}_{RMS}=\sqrt{\frac{{\sum_{i=0}^{N}\left(z_{t}^{original}-z_{i}^{mapped}\right)}^{2}}{N},}$$

where *N* is the total number of voxels inside the contoured right lung, and $z_{i}^{\text{original}}$ and $z_{i}^{\text{mapped}}$ represent the *z* coordinate for the *i*th voxel in the original and in the mapped image set respectively.

To investigate the contributions of other factors to the errors between the calculated and actual displacements, we carried out another optical flow calculation: We shifted the image set for 1 phase belonging to the 4D phantom by 8 slices in the superior–inferior direction, resulting in a superior placement. We then used optical flow to register the original image to the shifted image, and the resulting motion field to map the contour from the original image of the right lung to the shifted image. In this registration, the source and target images were identical with the exception of the imposed displacement. Thus, no 4D CT artifact was involved. Any error in the registration should result solely from the optical flow calculation itself.

**Figure 6:**
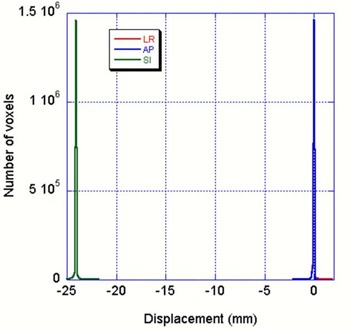
Histogram of the calculated displacement in contoured right lung based on the shifted phantom images of the thoracic phantom developed at the M.D. Anderson Radiological Physics Center. The shifted distance was 24 mm in the superior‐inferior (SI) direction. The lateral (LR) and anterior‐posterior (AP) directions both peaked sharply at 0 mm; the SI direction peaked at 24 mm. The full‐width half‐maximum values for all the peaks were about 0.2 mm.

Fig. 6 shows the histogram of the calculated displacement in the contoured right lung. The RMS superior‐inferior displacement is estimated at 23.9±0.1 mm for an actual distance shift of 24.0 mm. The difference is less than 0.1 mm. The bases of the peaks are also much smaller than those seen in Fig. 5. A comparison of Figs. 5 and 6 leads to the conclusion that the errors in the 4D CT data mapping came mainly from residual motion in the 4D CT scans and shaking of the motion table. Even with the errors not contributed by the optical flow calculation, the 0.7‐mm difference between the calculated mean error and reality for the 4D CT scans is quite reasonable, considering that the CT slice thickness is 3 mm.

Because of a lack of intensity variation inside the phantom lung, the aperture effect^(^ ^10^ ^,^ ^11^ ^)^ introduces some displacement error for some voxels. The less‐than‐0.1 mm difference between the calculated and the shifted motion distance in the Fig. 6 registration is caused by the aperture effect. Based on this analysis, the 4D CT artifact—that is, the residual motion—is the major error source. Improving 4D CT imaging would be the key to improving the registration.

**Figure 7:**
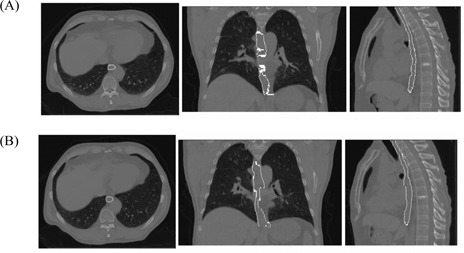
Esophagus contour from end‐expiration to end‐inspiration computed tomography (CT) images, mapped using optical flow. The two image sets were picked from the same location in the image coordinate system, not from exactly the same anatomic location. Panel A shows the original esophagus contour on end‐expiration CT. Panel B shows the mapped esophagus contour on end‐expiration CT.

**Figure 8:**
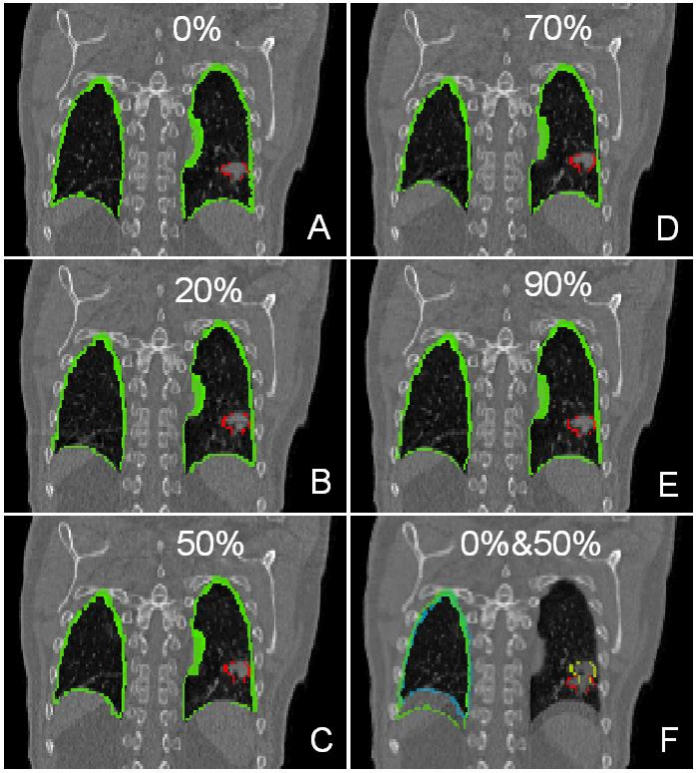
Coronal view of the contours of lungs and lung tumor mapped from end‐inspiration (0% phase) to all the other respiration phases using optical flow. The image sets shown in this figure are at the same location in image coordinate system, but not exactly the same location anatomically. The green lines represent the contours of the lungs while the red represents the gross tumor. Panel A shows the original contours drawn by a physician on the end‐inspiration phase, panel B, C, D and E are the mapped contours to the other phases, of which panel C is the end‐expiration phase (50% phase). Panel F shows the overlap of the end‐inspiration and end‐expiration phases (0% and 50% phases). The yellow contour is the original gross tumor contour on 0% phase, the red is the mapped contour on the 50% phase, while the blue is the original right lung on 0% and the dark green is the mapped right lung on the 50% phase.

For the CT images of actual patients, contours of lungs, liver, heart, esophagus, and gross tumor volume (GTV) are mapped from the end expiration image to the end inspiration image. Fig. 7 shows an example of the esophagus contour mapping. The registration for this figure used the parallel processing technique. Two computers ran the registration, with about half of the full image slices running on each computer. Fig. 8 shows an example of a 4D mapping series of lungs and a lung tumor. Note, in particular, the accuracy of the lung contour obtained without the use of threshold methods.

We compared a complete set of 4D CT images manually contoured for GTV with a corresponding set of images showing mapped GTV contours. The mapped contour series used as its starting point the GTV contour on end inspiration from the manually contoured series. Table 1 presents the displacements for the center of mass between the mapped and the manually contoured GTV over the respiration phases. The maximum displacement between the end expiration phase (50%) and the end inspiration phase (0%) is 1.3 cm for the mapped GTV and 1.5 cm for the manually contoured GTV. Direct measurement on a computer monitor of the CT images showed a similar range of motion.

**Table 1:** Displacement for the mapped (Map.) contours and the manually delineated (Man.) contours of the center of mass of the gross tumor volume^a^ over 10 respiration phases

	Lateral (mm)		AP (mm)		SI (mm)	
Phase (%)	Map.	Man.	Map.	Man.	Map.	Man.
0	0	0	0	0	0	0
10	0	−1	0	1	0	0
20	1	1	0	1	3	6
30	0	2	0	0	9	12
40	2	3	−1	1	12	15
50	4	3	0	0	12	15
60	2	3	−1	−1	12	15
70	2	2	0	−1	9	12
80	0	1	0	0	6	6
90	0	0	0	1	0	3

a The location of the gross tumor volume at the end inspiration phase (0%) is set at 0 (lateral), 0 [anterior–posterior (AP)], and 0 [superior–inferior (SI)]. The 50% phase is end expiration, where maximum displacement is observed. The displacement is mainly in the SI direction.

We also calculated the volume variation of the GTV over the full respiration cycle. The volume of the manually contoured GTV was $12.3\pm 0.6\;{\text{cm}}^{3}$. The mapped GTV volume was $12.5\pm 0.5\;{\text{cm}}^{3}$, another demonstration that the GTV mapping was stable and reliable, considering that the motion range was relatively large, but that the volume change was small. Also, the volume of the mapped GTV was slightly more consistent than that of the manually contoured GTV over the respiration cycle.

We presented the 3D contours of esophagus, lungs, GTV, and heart over the multiple respiration phases to clinicians, including 3 physicians, 3 physicists, and 3 dosimetrists. The clinicians were asked if they needed to change any part of the contours. In more than 90% of cases, the clinicians said that no changes were needed. For a few cases, the clinicians made small changes. Some changes were made on the original contours (which had nothing to do with the optical flow mapping). However, those changes were insignificant. The volume changes attributable to the contour changes all represented less than 10% of the original volume.

The aperture effect in optical flow algorithms introduces errors in local volumes where intensity variation is lacking. Although these errors are seldom a serious problem in 3D contour mapping because the contours are usually sitting at the edges, where intensity changes more significantly than in the surrounding volumes, they reduce the accuracy of the optical flow calculation in mapping a volume of interest in which local flat intensity regions are found. The problem of aperture effect would be more significant if the images were noisier. Improving 4D CT imaging would assist in reducing aperture effect.

It has been proved that the aperture problem can be resolved by introducing a second or even higher‐order intensity derivative into the optical flow constraint equations.^(12)^ The 3D optical flow program applied in the present study uses only first‐order intensity derivatives in the optical flow equations. There is room to improve the accuracy by using higher‐order intensity derivatives when necessary. The tradeoff to that approach would be a slowdown in the calculation.

Image registration consumes a great deal of time in addition to a great deal of memory. The multi‐resolution feature not only enhances the accuracy of the registration, but also significantly reduces the calculation time. When, after registration of the coarser resolution, registration of the finest resolution commences, the deformation is already close to the target image. Thus, only a few iterations are needed to reach an acceptable registration. Without multi‐resolution, the number of iterations needed is usually approximately 100. For registration of 512×512×70 voxels per image set, the multi‐resolution feature reduces the calculation time from about 10 hours to about 20 minutes on a personal computer with a 2.66 GHz processor and 4 GB of memory. The computation time is further reduced to less than 5 minutes if the final motion field is solely linear interpolated from a lower‐resolution registration—a situation that also reduces the precision of registration by a factor of 2, however.

## IV. CONCLUSIONS

A deformable image registration matrix, describing the deformation of a 3D CT image data set from one phase of the respiratory cycle to the other, obtained by use of an optical flow algorithm can be used to generate a set of contours of normal anatomic structures and the GTV in all phases of a 4D CT image data set.

## Supporting information

Supplementary Material
