# Supplementary figures and images for: Use of three‐dimensional (3D) optical flow method in mapping 3D anatomic structure and tumor contours across four‐dimensional computed tomography data
Source: J Appl Clin Med Phys. 2008 Feb 5;9(1):59–69. doi: 10.1120/jacmp.v9i1.2738 (PMC5721534; doi:10.1120/jacmp.v9i1.2738)

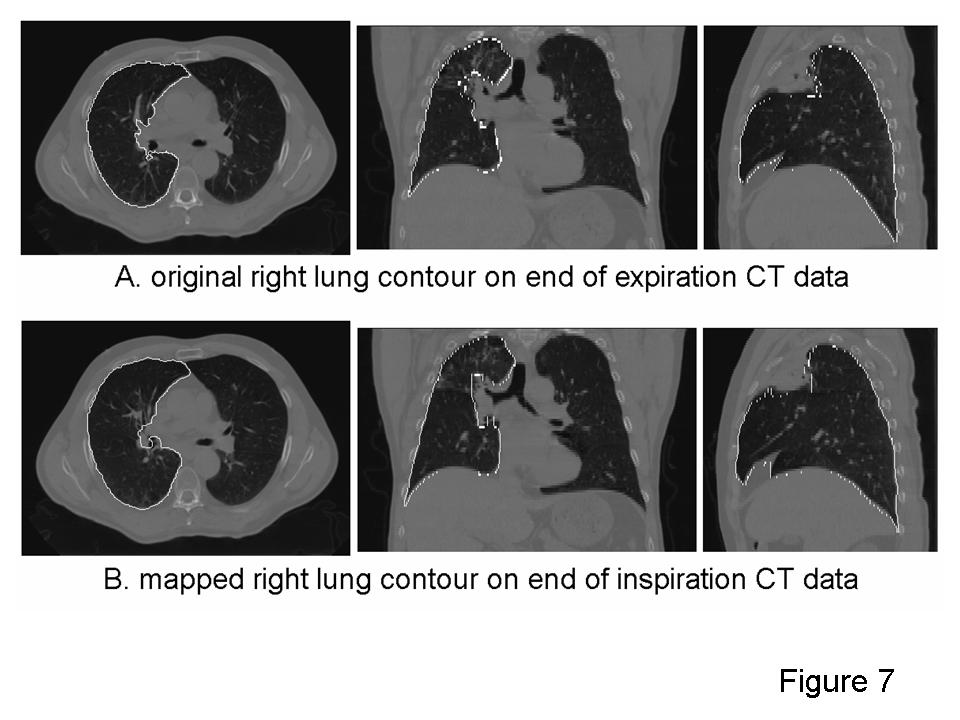

Supplement: Supplementary file 1 — Supplementary Material [file ACM2-9-59-s001.jpg]
